# Extracellular vesicle miRNome during subclinical mastitis in dairy cows

**DOI:** 10.1186/s13567-024-01367-x

**Published:** 2024-09-19

**Authors:** Matteo Cuccato, Sara Divari, Diana Giannuzzi, Cristina Grange, Riccardo Moretti, Andrea Rinaldi, Christine Leroux, Paola Sacchi, Francesca Tiziana Cannizzo

**Affiliations:** 1 Department of Veterinary Sciences, University of Turin, Grugliasco, 10095 Turin, Italy; 2 Department of Agronomy, Food, Natural Resources, Animals and Environment, University of Padua, 35020 Padua, Italy; 3 Department of Medical Sciences, VEXTRA Facility, University of Turin, 10126 Turin, Italy; 4 Faculty of Biomedical Sciences, Institute of Oncology Research (IOR), Università della Svizzera Italiana (USI), 6500 Bellinzona, Switzerland; 5 Université Clermont Auvergne, INRAE, VetAgro Sup, UMR Herbivores, 63122 Saint-Genès-Champanelle, France; 6 Department of Food Science and Technology, University of California Davis, Davis, CA USA

**Keywords:** Mastitis, bovine, dairy cows, microRNA, extracellular vesicles, small RNA-seq

## Abstract

Bovine mastitis is one of the main inflammatory diseases that can affect the udder during lactation. Somatic cell counts and sometimes microbiological tests are routinely adopted during monitoring diagnostics in dairy herds. However, subclinical mastitis is challenging to identify, reducing the possibility of early treatments. The main aim of this study was to investigate the miRNome profile of extracellular vesicles isolated from milk as potential biomarkers of subclinical mastitis. Milk samples were collected from a total of 60 dairy cows during routine monitoring tests. Small RNA sequencing technology was applied to extracellular vesicles of milk samples collected from cows classified according to the somatic cell count to identify differences in the miRNome between mastitic and healthy cows. A total of 1997 miRNAs were differentially expressed between both groups. Among them, 68 miRNAs whose FDRs were < 0.05 were mostly downregulated, with only one upregulated miRNA (i.e., *miR*-361). Functional analysis revealed that *miR*-455-3p, *miR*-503-3p, *miR*-1301-3p and *miR*-361-5p are involved in the regulation of several biological processes related to mastitis, including immune system-related processes. This study suggests the involvement of extracellular vesicle-derived miRNAs in the regulation of mastitis. Moreover, these findings provide evidence that miRNAs from milk extracellular vesicles can be used to identify biomarkers of mastitis. However, further studies must be conducted to validate these miRNAs, especially for subclinical diagnosis.

**Supplementary Information:**

The online version contains supplementary material available at 10.1186/s13567-024-01367-x.

## Introduction

Bovine mastitis is the principal cause of economic losses in the dairy industry due to milk production and quality reduction [1]. In addition, dairy farmers have to address increased costs for treatments and the increased culling rates caused by mastitis outbreaks [2]. The udder infection is caused mostly by a variety of microorganisms, mainly bacteria (*Staphylococcus* spp., *Streptococcus* spp., and *Enterobacteriaceae*), but also by yeast belonging to *Candida* spp. or protozoa of the genus *Prototheca* [3]. The clinical classification of mastitis differentiates the disease into clinical and subclinical forms according to the presence or absence of symptoms and signs, such as visibly abnormal milk, swelling, heat, pain and redness of the udder [3]. Subclinical forms are the main challenge for mastitis control due to the normal presentation of both the udder and milk, but with increased somatic cell count (SCC) and the presence of bacteria in milk [4]. According to recent literature on the prevalence of bovine mastitis worldwide, subclinical mastitis is the most prevalent and causes major economic losses, with an estimated value of 2 billion USD per year [5–7]. In particular, in Europe, the prevalence of subclinical mastitis is ∼37% [5], whereas in Italy, it is ∼22.5% [8]. Therefore, the early identification of subclinical forms is fundamental for adequate treatment and sustainable dairy herd management. Furthermore, with Regulation (EU) 2019/6 of the European Parliament about veterinary medicinal products, the European Union introduced strict limitations on the use of antimicrobials for prophylaxis and metaphylaxis purposes to control the rise and spread of resistance phenomena. In the past, the preventive use of antimicrobials in dairy herds was frequently adopted, especially during the dry period [1, 9]. In the new regulatory scenario, veterinary practitioners working in the dairy industry require alternative solutions for the control of mastitis, including genomic selection for resistance [10] and novel diagnostic and therapeutic tools [1].

Monitoring subclinical mastitis is an essential requirement in dairy herd management and consists of SCC measurements and microbiological tests [3]. Moreover, the SCC parameter is also considered to define milk suitability for human consumption and quality for cheese processing; therefore, milk pricing strictly depends on the SCC [9]. Unanimous agreement considers milk with an SCC value greater than 400 000 cells/mL, regardless of the presence of clinical symptoms, as mastitic [2, 11]. On the other hand, milk from a healthy udder has an SCC value lower than 100 000 cells/mL [2, 9]. An SCC measurement between those values should be interpreted: it could be typical of subclinical mastitis or a finding related to the recovery processes of the mammary gland after infection, when inflammation and pathogens could still be found [11, 12]. Currently, an SCC value equal to 200 000 cells/mL has been adopted as a threshold to diagnose subclinical mastitis [13]. However, inflammation of the mammary gland has also been observed at values of ∼100 000 cells/mL, especially in primiparous cows [14]. Thus, new biomarkers would help to discriminate cows with subclinical mastitis.

In recent years, extracellular vesicles (EVs) have been widely investigated in human and veterinary medicine. EVs are membrane-limited nanoparticles involved in intercellular communication, and their presence has been proven in several biological fluids, such as blood, urine, milk, and saliva [15–17].

Among EVs, three main classes are included: exosomes, ectosomes and apoptotic bodies [18]. Classes are defined according to the range size of EVs and are divided into small-sized EVs (<200 nm) and medium/large-sized EVs (>200 nm) [19]. Owing to their biogenesis, exosomes can be generated through the endosomal complex required for transport (ESCRT) or the ceramide-dependent pathway. The exosome size is normally lower than 100 nm [20]. In contrast, ectosomes originate through exocytosis from the cell membrane. In this case, the dimensions are larger than those of exosomes, and their size can reach 1000 nm [21]. Finally, the classification of apoptotic bodies into EVs is still under debate, and they are generated during apoptosis and are highly variable in size [19].

The ability of EVs to regulate cellular and organ processes is due to the presence of different types of cargo inside EVs, such as noncoding RNA, mRNA, DNA, proteins and lipids, which can be delivered to the targeted recipient cell [20]. The ability of EVs to regulate cellular communication also relies on the presence of several protein and glycoprote in membrane markers [22]. In the last decade, EVs have been largely studied and evaluated as innovative biomarkers in human medicine, particularly for cancer and neurodegenerative diseases.

In veterinary medicine, the role of microRNAs (miRNAs) has been particularly investigated in mastitis pathogenesis among domesticated ruminant species (i.e., cows, sheep and goats). miRNAs are an extremely important group of small noncoding RNAs, ranging in length from 20 to 22 nucleotides, that regulate gene expression through mRNA silencing at the posttranscriptional level [23]. Several studies have been conducted in experimentally infected cows, and several putative miRNAs that are differentially regulated, mainly under *Staphylococcus aureus* or *Escherichia coli* infection, have been identified [24–26]. Similar to other infectious diseases, during mastitis, miRNAs are involved in the regulation of several pathways, such as pathogen response, activation and regulation of inflammatory processes and regulation of the immune response [27]. The identification of early indicators for rapid and accurate detection of mastitis could lead to earlier and more effective treatment, allowing the animal to recover faster and therefore reducing the associated economic losses. In addition, a better understanding of the molecular regulation of the mammary response to inflammation would allow the identification of such robust indicators. In this context, the main aims of this study were to investigate the role of miRNAs carried by EVs in the regulation of mastitis and to identify putative biomarkers for the early diagnosis of mastitis.

## Materials and methods

### Study design and sample collection

As previously described [28], a total of 120 primiparous Holstein Friesian cows belonging to 10 dairy farms located in the provinces of Cuneo and Turin (Piedmont Region, Northern Italy) were selected without any clinical signs of disease. Animals were managed according to local farm production practices. All manipulations were performed according to the best animal handling and veterinary practices to avoid animal distress. Before sample collection, the teats were disinfected, and the first milk ejected was collected in a specific container and then discarded. Individual samples were collected in sterile polypropylene tubes (2 aliquots of 50 mL) from all quarters of each cow and immediately stored at 4 °C. Before EV isolation and sequencing, a total of 60 milk samples were randomly selected and further processed for EV isolation. Milk sampling was conducted during the winter (December 2020–February 2021, *n* = 52) and summer (June 2021–September 2021, *n* = 8) seasons. Before sequencing, samples were clustered using an SCC threshold level of 200 000 cells/mL, normally considered the SCC level of subclinical mastitis, into two groups: high SCC (group H, *n* = 11) and low SCC (group L, *n* = 49). The milk samples in the two groups are shown in Table 1.

**Table 1:** **Milk parameters of the high (H) and low (L) somatic cell count (SCC) groups**

	Group H (*n* = 11)	Group L (*n* = 49)
SCC (mean cells/mL ± sd)	1.75 × 10^+6^ ± 2.13 × 10^+6^	4.35 × 10^+4^ ± 4.71 × 10^+4^
DIM (mean days ± sd)	197.27 ± 119.29	124.43 ± 74.45
*Staphylococcus* spp. positivity	2	17
*Streptococcus uberis* positivity	2	Not detected
Negative for bacteriology	7	32
Age (mean years ± sd)	2.77 ± 0.41	2.68 ± 0.41

DIM: days in milk, sd: standard deviation.

### EVs isolation

Milk aliquots were processed with consecutive centrifugations, whose steps were as follows: 3000 × *g* for 10 min at 4 °C to remove milk fat and somatic cells, an additional step of 3000 × *g* for 10 min at 4 °C to remove additional fat and cell residuals, 5000 × *g* for 30 min at 4 °C to remove larger cell debris and finally 10 000 × *g* for 30 min at 4 °C to remove smaller cell debris. After these centrifugation steps, the skim milk was stored at −80 °C until further analyses.

Then, the skim milk samples were thawed gradually on ice. EVs isolation was performed by size exclusion chromatography (SEC), in detail, with the original 70 mm columns of qEV (Izon Science, Lyon, France). Before loading milk samples on the SEC column, the starting volume of 4 mL was reduced using a centrifugation filter tube (AMICON ULTRA-4 50 kDa, Merck Millipore, Burlington, MA, USA) to reach a volume of 500 µL, the maximal loadable capacity of the SEC column. Centrifugation was performed at 4000 × *g* at 4 °C for 40 min to 60 min, depending on the viscosity of the sample. To avoid protein precipitation at the filter level, during centrifugation, samples were gently mixed by slow pipetting on ice every 5–10 min pausing the centrifugation. Once the volume reached 500 µL, milk samples were loaded on the SEC column, which was previously mounted on its automatic fraction collector (qEV AFC system, Izon Science). Following the manufacturer’s instructions, the first five aliquots (50 µL each) were separately collected in 2 mL tubes corresponding to the most rich and pure EVs fractions. The five EVs fractions (250 µL) were subsequently mixed, and the volume was reduced to reach 50 µL, the minimal useful volume for downstream applications, via a centrifugation filter tube (AMICON ULTRA-4 50 kDa, Merck Millipore). Centrifugation was performed at 4000 × *g* at 4 °C with a variable time from 15 min to 30 min depending on the sample viscosity. To avoid protein precipitation at the filter level, during centrifugation, samples were gently mixed by slow pipetting on ice every 5–10 min pausing the centrifugation. Finally, the concentrated EVs were stored at -80 °C until further analyses.

### EVs validation

#### Nanoparticle tracking analysis (NTA)

EV concentrations were calculated via nanoparticle tracking analysis (NTA) using the Nanosight LS300 system (Malvern Panalytical, Malvern, UK) equipped with a 488 nm laser. EVs were diluted (1:200) in 0.1 μm filtered saline solution and analysed via NTA 3.2 Analytical Software. Three videos of 60 s at camera level 15 and threshold 5 were captured via a syringe pump 50. The settings were kept constant between samples.

### Transmission electron microscopy (TEM)

Transmission electron microscopy (TEM) was performed to evaluate EV integrity and size. EVs were fixed on 200 mesh nickel formvar carbon-coated grids (Electron Microscopy Science, Hatfield, PA, USA) as reported by Bruno et al. [29]. EVs were negatively stained using NanoVan™ and Nano-W™ (Nanoprobes, Yaphank, NY, USA) and observed using a Jeol JEM 1010 electron microscope (Jeol, Tokyo, Japan).

### Western blot

To validate the EV isolation method and confirm the presence of EVs in the samples, western blot analysis was conducted. In particular, three EV markers were selected as suggested by the MISEV guidelines: TSG101, CD9 and calnexin [19]. Total proteins were extracted from EV lysates using RIPA buffer supplemented with a protease inhibitor cocktail (Sigma‒Aldrich, St. Louis, MO, USA) and quantified via a DC protein assay (Bio-Rad, Hercules, CA, USA). Equal amounts of protein (300 µg/lane) were resolved with 4–20% MP TGX Stain-Free Gel (Bio-Rad) under reducing conditions for only TSG101 and Calnexin. Proteins were blotted onto PVDF membranes (Trans-Blot Turbo Mini PVDF, Bio-Rad) using Mini Trans-Blot cells (Bio-Rad). The blotted membranes were blocked with a 10% BSA solution (Merck Millipore) for 1 h at room temperature, followed by an overnight incubation at 4 °C with a mouse monoclonal anti-TSG101 primary antibody (1:200; sc-7964, Santa Cruz Biotechnology, Dallas, TX, USA), a mouse monoclonal anti-CD9 primary antibody (1:500; MCA469GT, Bio-Rad) and a rabbit polyclonal anti-calnexin antibody (1:200, sc-11397, Santa Cruz Biotechnology). The membranes were subsequently incubated with secondary horseradish peroxidase (HRP)-conjugated anti-mouse (1:10 000) and anti-rabbit (1:40 000) antibodies and developed with Clarity Western ECL Substrate (Bio-Rad). The immunoblot bands were visualized via the ChemiDoc MP System (Bio-Rad). For western blot analysis, a ready-to-use protein extract from A-431 cell lysate (sc-2201, Santa Cruz Biotechnology) was used as a positive control for calnexin.

### Small RNAs extraction

Total small RNAs were extracted from EV samples via a Maxwell RSC miRNA Blood Kit (Promega, Madison, WI, USA) following the manufacturer’s instructions. The input miRNAs were subsequently quantified using the Qubit microRNA Assay Kit. Finally, the small RNA profiles of the input RNA samples were investigated via the Agilent small RNA kit for the Bioanalyzer 2100 instrument (Agilent Technologies, Santa Clara, CA, USA).

### Small RNA sequencing

Library preparation for next-generation sequencing was performed using SMARTer smRNA-seq kit for Illumina (Cat. no. #635031; Clontech Laboratories Inc., Kusatsu, Japan) according to the manufacturer’s protocol. Following PCR amplification, purification, and validation, the size selection of the sequencing libraries was performed using SPRIselect beads (Cat. no. #B23318, Beckman Coulter Life Science, Brea, CA, USA). Quality controls were performed on a Bioanalyzer 2100 (Agilent Technologies) and Qubit V4 (Thermo Fisher Scientific). Next-generation sequencing was performed on a NextSeq500 (Illumina, San Diego, CA, USA) with the reagents kit V2 (75 cycles; Illumina). The samples were processed starting from single-ended 75 bp-long sequencing reads.

### Bioinformatic analysis

The FASTQ files were trimmed using cutadapt (cutadapt 3.5 with Python 3.7.7) following the manufacturer’s instructions (parameters: -m 15 -u 3 -a AAAAAAAAAA). Trimmed fastq files were processed using the miRNAseq workflow implemented in Docker4seq [30–32]. Briefly, quality control of trimmed reads was performed using the FastQC software v. 0.11.9 [33]. The quality of the trimmed reads was checked to evaluate the overall distribution of sequenced fragment length. The trimmed reads were subsequently mapped onto bovine miRNA precursors (miRbase 22) using the BWA aligner (v. 0.7.12) [34], and mature 5p and 3p miRNAs were counted using an R script embedded in the docker4seq workflow.

Count filtering, data normalization, and differential expression analysis were performed in RStudio. We first normalized the miRNA count matrix with the sequencing depth for each sample by calculating counts per million (CPM). Then, we filtered out genes expressed in fewer than 10 samples with CPM < 0.5 via the *cpm()* function from the edgeR package (v. 3.36.0) [35]. miRNAs failing these criteria were removed from the count matrix before exploration and differential expression analyses.

Once a filtered miRNA matrix was obtained, exploratory analysis of the expressed miRNAs was performed via unsupervised principal component analysis (PCA) and nonparametric multidimensional similarity (NMDS) analysis with the ggplot2 (v. 3.3.5) R package [36]. Differentially expressed (DE) miRNA analysis was performed pairwise via the edgeR (v. 3.36.0) package [35]. Differential analysis was performed by comparing samples according to their SCC threshold level of 200 000 cells/mL. Counts from expressed genes were first normalized with the *calcNormFactors()* function [37]. The *voom()* function from the limma R package (v. 3.50.0) was subsequently used to fit a generalized linear regression model to correct the data with the group as a fixed effect [38]. The *p* values were adjusted for multiple testing via the Benjamini and Hochberg procedure [39]. Only DE miRNAs with an adjusted *P* value < 0.05 were used for downstream pathway analysis.

### In silico functional analysis

Using only DE miRNAs, a predictive functional analysis was performed to evaluate the influence of the DE miRNAs on biological processes and pathways. In silico analyses were performed using human orthologues (*hsa-*), instead of bovine orthologues (*bta-*), since information about miRNA activity is more detailed and extensive in humans than in bovines. In this particular step, we used miRWalk 2.0 [40], OmicsNet 2.0 [41] and Cytoscape v.3.9.1 with the ClueGO plugin v.2.5.9 [42] software. First, the most deposited mature forms (-3p or -5p) of DE miRNAs were checked in the miRBase database and modified accordingly in the DE miRNA list with the aim of retrieving the most relevant results via functional analysis. The partially modified DE miRNA list was subsequently used in miRWalk to retrieve all the miRNA-gene interactions. In particular, only validated interactions deposited in the miRTarBase database and with biding position data (related to the 3’UTR, 5’UTR and CDS) were selected. In parallel, to identify functional biological categories related to DE miRNAs, Panther biological process analysis was performed via OmicsNet. Then, the categorization of biological processes was manually performed. Biological processes were clustered into two main macro-categories: cell life processes (including cell cycle, regulation of the cell cycle, cell proliferation and apoptotic process) and gene expression machinery processes (including regulation of transcription by RNA polymerase II, transcription DNA templating, mRNA processing, mRNA splicing via the spliceosome, and regulation of translation and protein phosphorylation). In addition, target genes involved in the regulation of immune processes were obtained with the ClueGO plugin in Cytoscape via the function GO-ImmuneSystemProcess. These gene lists were filtered by selecting only validated miRNA-gene interactions previously obtained via miRWalk. The miRNAs-genes network was visualized via Cytoscape, which was subsequently used to identify potential networks.

## Results

### EV validation by NTA, TEM and Western blot analyses

**Figure 1:**
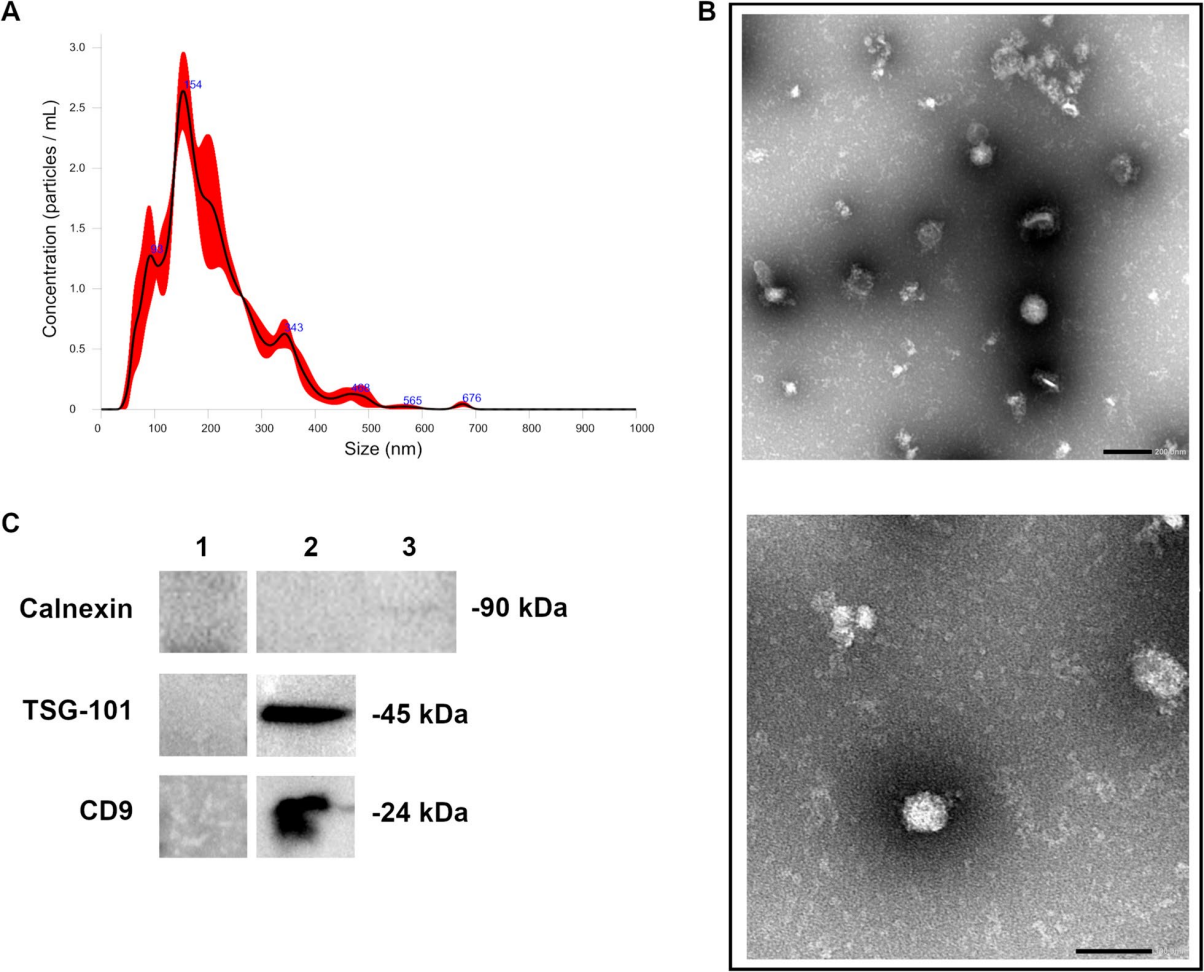
**EV validation.** Representative graphs of the results of nanoparticle tracking analysis (NTA) showing EV size distribution (**A**). Representative TEM images of EVs showing intact and heterogeneous EVs; top and bottom scale bars, 200 nm and 100 nm, respectively (**B**). Western blot analysis of EV samples. 1: isotype control antibody on EVs, 2: EVs, 3: cell lysate (A-431 cell line) (**C**). The EV markers used for analysis were TSG-101 (∼45 kDa), CD9 (∼24 kDa) and calnexin (∼90 kDa).

To validate the presence of EVs in the samples, NTA revealed an average size distribution of EVs of 171.87 ± 33.93 nm (Figure 1A), and TEM analysis (Figure 1B) confirmed that the EVs had a homogeneous pattern of nanosized membrane vesicles. Moreover, western blot analysis was performed on representative samples randomly selected from the collected samples. Both EV markers, TSG101 and CD9, were positive in the samples analysed, whereas calnexin was negative in the EV samples. The specific bands of the two targets are reported in Figure 1C.

### Differential analyses of miRNAs

**Figure 2:**
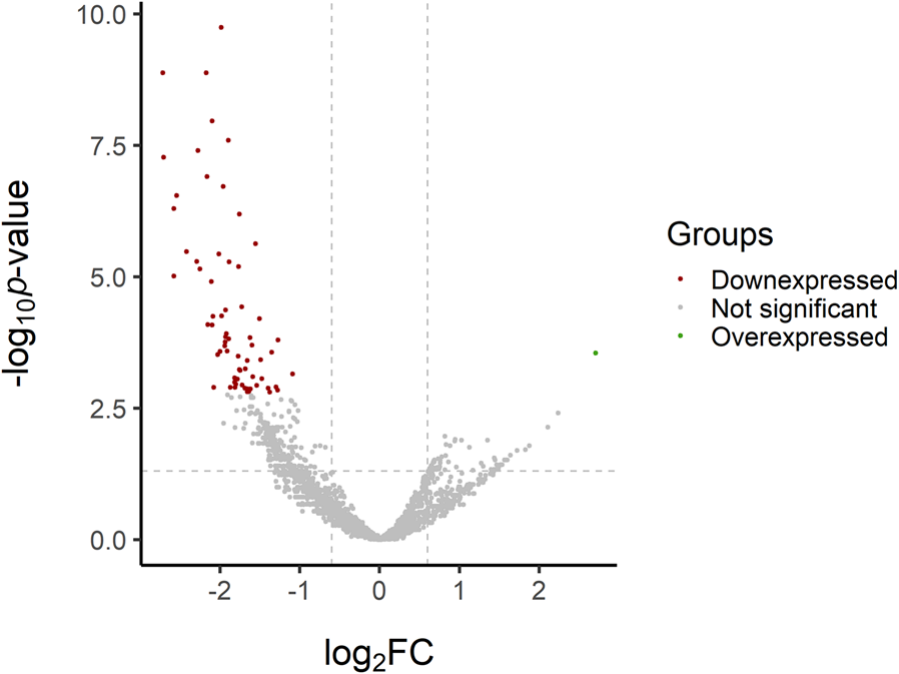
**Differentially expressed miRNAs between group H and group L.** The regulation of expression is intended to be downregulated/upregulated in group H in comparison with that in group L.

In total, 2127 *Bos taurus*-annotated miRNAs (bta-miRNAs) were detected. A PCA table was generated to investigate the sample distribution (Additional file 1). Using a cut-off value for the SCC parameter of 200 000 cells/mL, a total of 68 miRNAs were differentially expressed (DE) with a false discovery rate (FDR) < 0.05 between group H and group L (miRNAs were up-/downregulated in group H in comparison to group L). These genes were mostly downregulated. Intriguingly, only 1 miRNA, *bta*-*miR*-361-3p, was upregulated, with a 2.7-fold change (FC) value. Among the downregulated miRNAs, 15 had a FC < −2. A volcano plot of the DE miRNAs is shown in Figure 2. The list of the 68 DE miRNAs is reported in Additional file 2.

### Functional analysis of DE miRNAs

**Figure 3:**
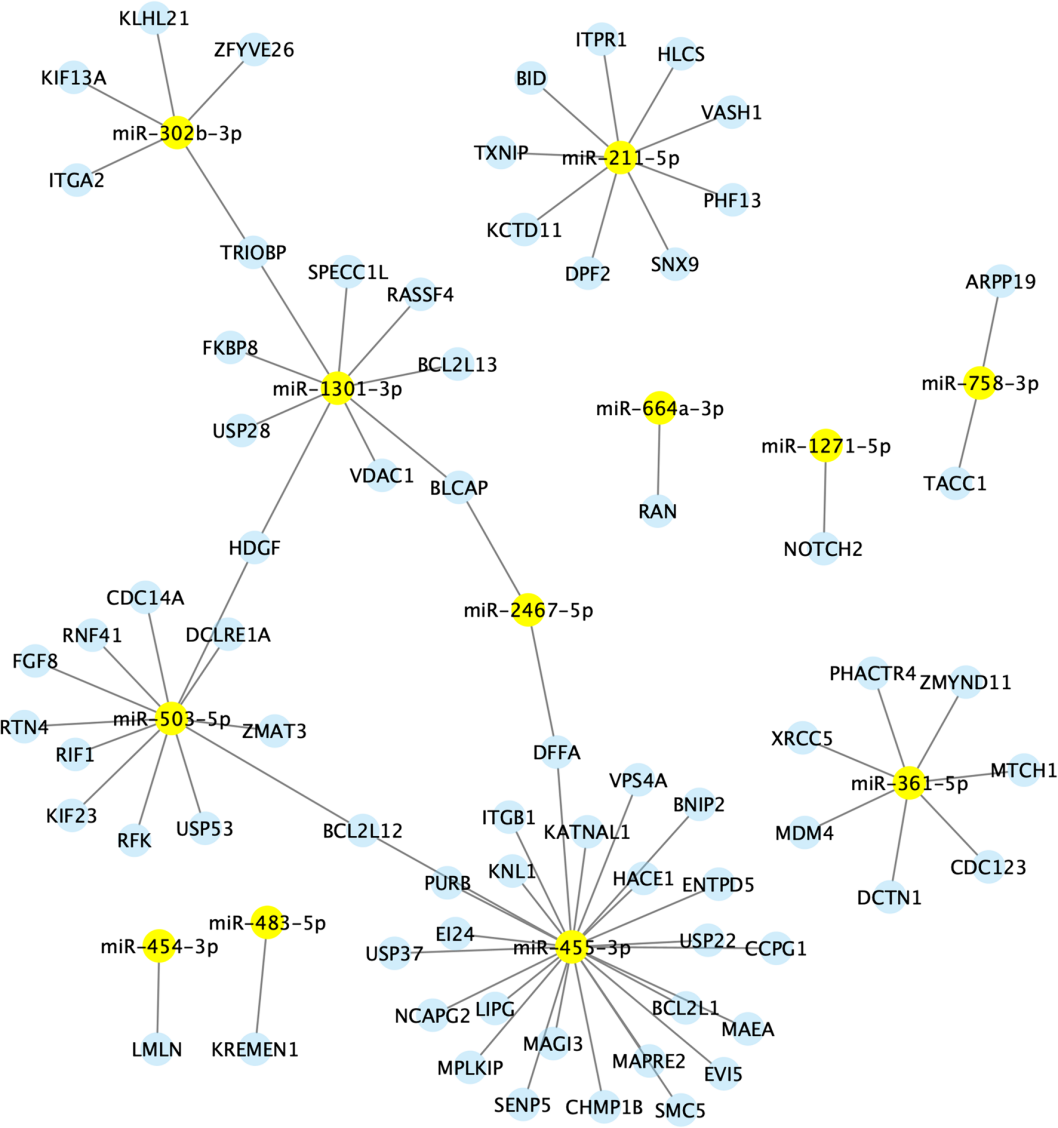
**Networks of the differentially abundant miRNAs identified via Cytoscape.** These results are related to miRNA-gene interactions involved in cell life processes.

**Figure 4:**
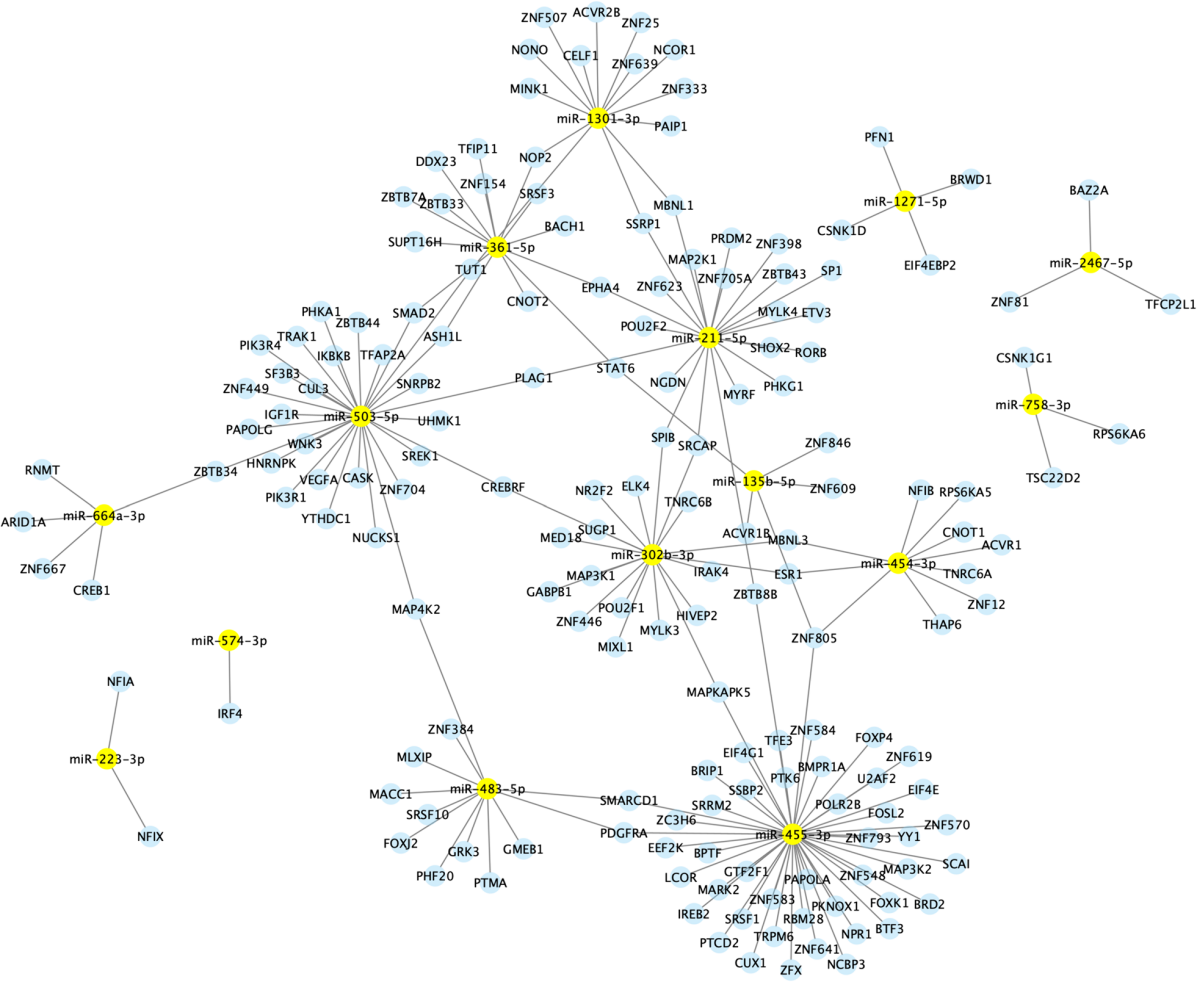
**Networks of the differentially abundant miRNAs identified via Cytoscape**. These results are related to the miRNA-gene interactions involved in the gene expression machinery.

**Figure 5:**
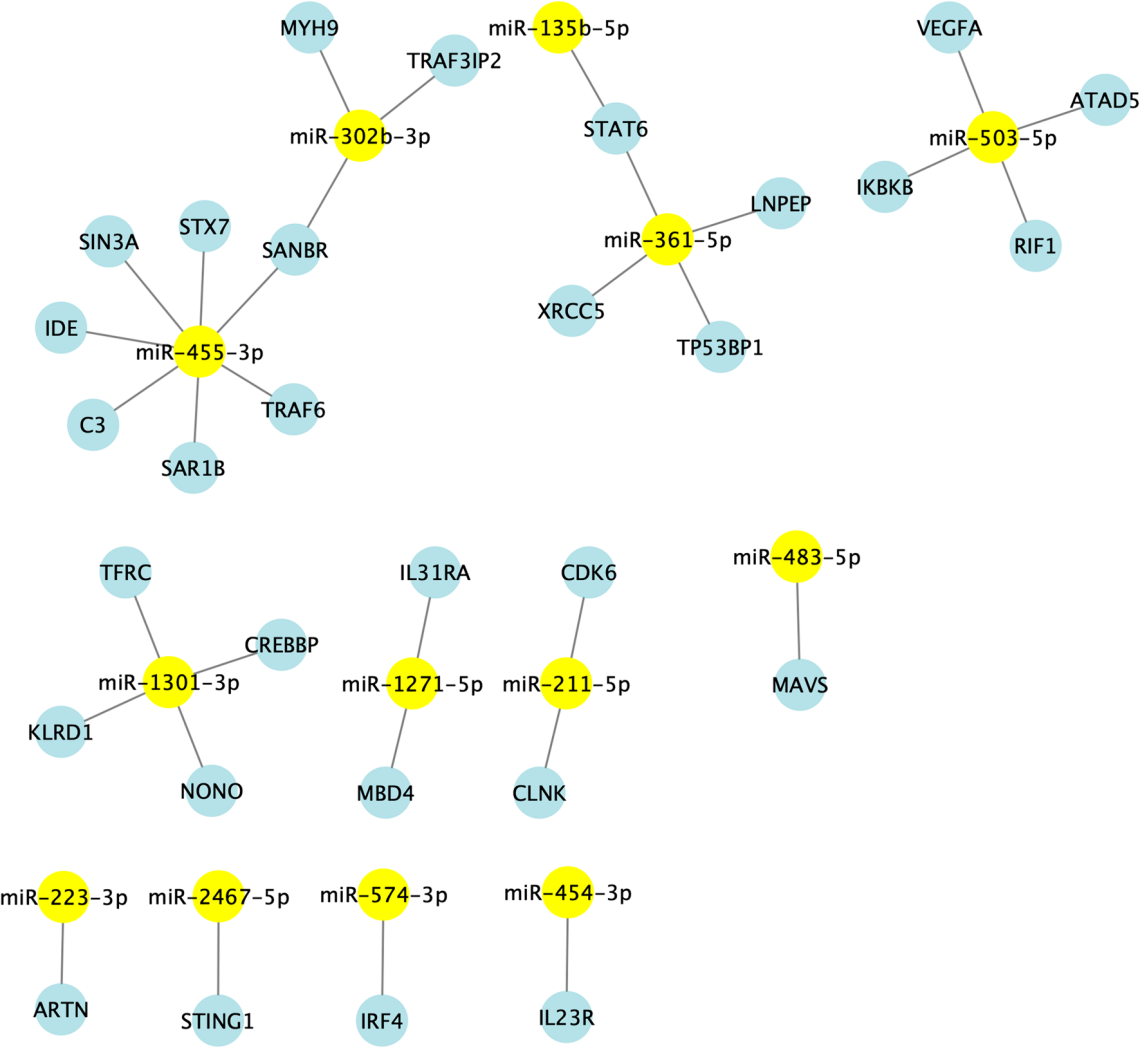
**Networks of the differentially abundant miRNAs identified via Cytoscape.** These results are related to miRNA-gene interactions involved in immune system processes.

The functional analysis was conducted using human orthologues (*hsa*-) instead of bovine orthologues (*bta*-) since human databases are more complete and more informative regarding miRNA function. The targets of 17 known miRNAs were obtained from OmicsNet via the Panther database. In addition, the regulation of immune system processes was investigated via the ClueGO plugin in Cytoscape. The main immune processes significantly influenced by the DE miRNAs were related mainly to mediated immunity, including immunoglobulin production and lymphocyte regulation. Therefore, the miRNA-gene networks were visualized via Cytoscape, and three separate reactomes were generated considering cell life (Figure 2), gene expression (Figure 3) and immune processes (Figure 4). Two large nodes involved in the regulation of miRNA-gene interactions are *miR*-503-5p and *miR*-455-3p, which are involved in both cell life and gene expression processes. The third network related to immunity is less complex than the other two networks are. However, 4 main highlighted miRNAs (*miR*-455-3p, *miR*-503-3p, *miR*-1301-3p and *miR*-361-5p) were involved in the regulation of immunity (Figure 5).

## Discussion

Bovine mastitis is one of the main challenges that farmers and veterinarians routinely face in dairy farming. Moreover, the spread of antimicrobial resistance and the recent enactment of the new European Regulation about the use of veterinary medicinal products have increased the need for the implementation of diagnostic tools to detect subclinical mastitis early to reduce/avoid antimicrobial treatments [43]. Among the different parameters of mammary gland infection, an increase in the SCC in milk is considered to have prognostic value. Therefore, to compare healthy subjects with cows potentially affected by subclinical mastitis, the milk samples were classified according to the SCC measurement, setting the 200 000 cell/mL value as a threshold [9]. In this study, the miRNome profile of dairy cows affected by subclinical mastitis was investigated and compared with that of healthy control samples. EVs were obtained from milk samples to better understand the role of miRNAs in the regulation of mastitis and to identify new potential biomarkers of the disease. EVs and miRNAs have been largely studied in human and veterinary medicine for their evaluation as potential biomarkers of different diseases, such as cancer and neurodegenerative and infectious diseases. In this study, individual milk samples were collected from a total of 60 Holstein Friesian cows during routine mastitis screening tests and subjected to EV small RNA-seq analysis to detect miRNome profiles suggestive of the subclinical form of the disease. Indeed, bovine mastitis can modify the milk miRNome, and several miRNAs have been reported to be differentially expressed during mammary gland inflammation [23].

The results of the EV miRNome profiling revealed statistically significant differences between samples with high SCC levels (>200 000 cell/mL, group H, considered a group with subclinical mastitis) and samples with low SCC levels (<200 000 cell/mL, group L, considered a healthy control group). In particular, 68 miRNAs were DE, most of which were downregulated in group H compared with group L. Among these 68 DE miRNAs, 15 miRNAs were downregulated, with FC values < **-**2. Many of these DE miRNAs were specifically related to bovine species, and no relevant information could be retrieved from the scientific literature. However, three miRNAs can be highlighted: *miR*-223, *miR*-124 and *miR-*568. The first one, i.e., *miR*-223, is largely described in the literature as regulating the inflammatory response in bovine mastitis [44–48]. All these studies reported the upregulation of *miR*-223 in response to *S. aureus* and *S. uberis*. or *S. agalactiae* experimental infections. Therefore, the finding of *miR*-223 downregulation in the present study is not consistent with what has been reported in the scientific literature. One possible explanation for this controversial result may be related to the different matrices used for miRNA extraction. The abovementioned studies investigated the role of miRNAs in different mastitis models involving mammary gland biopsies [44, 46, 47], cultured Mac-T cells [24] or blood [45]. Therefore, an adequate comparison is not possible. Moreover, in the dataset of this study, only two samples were positive for *S. uberis*, which may not be enough to highlight differences between the groups. In contrast, Tzelos and colleagues investigated the expression of miR-223 during mastitis, but a statistically significant difference in expression was not detected between mastitic and healthy cows [49]. In addition, in Tzelos’s study, miR-223 was investigated both in whole and skim milk samples and not in milk EVs, affecting the possibility of an adequate comparison. However, according to the human medical literature, *miR*-223 is either expressed almost exclusively or highly enriched in several subsets of white blood cells, including T- and B-lymphocytes, neutrophils, mast cells and monocytes [50]. It is well known that miRNAs that can be detected in milk can have different origins according to the different cells composing and characterizing the mammary gland during mastitis, i.e., mammary epithelial cells, inflammatory cells, adipocytes, fibroblasts or myoepithelial cells [36]. On the other hand, *miR*-124 and *miR*-568 were also significantly downregulated in this study and, for the first time, were reported to be related to bovine mastitis. According to the literature, *miR*-124 has been described to regulate the parasitic pathogenesis of *Schistosoma japonicum* and *Fasciola gigantica* in both bovines and buffaloes [51–53]. Intriguingly, the inhibitory effect of *mi*R-568 on CD4+ T cells was previously demonstrated, suggesting a role in the modulation of lymphocyte activity [54].

According to the functional analysis, four miRNAs (namely, *miR*-455, *miR*-361, *miR-*1301, and *miR-*503) were found to be involved in the regulation of the biological processes of gene expression, cell life and the immune response. As mentioned previously, the functional analysis was conducted using human orthologues (*hsa*-) rather than bovine orthologues (*bta*-). Given this possible bias, the functional results obtained in this study may be considered predictive, and further studies must be conducted to validate these predictions. However, the results can be compared with the scientific literature to contextualize and explain the roles of these four miRNAs. *MiR-*455 has already been reported in dairy cows subjected to dietary regulation. For example, Webb and colleagues reported a downregulation of *miR-*455 in the period after calving in cows fed a highly balanced diet [55]. Our results are consistent with those of this study since both the downregulation of *miR*-455 and its involvement in the regulation of the inflammatory response have been reported. According to the literature in humans, *miR-*455 downregulation is associated with the activation of inflammatory pathways in multiple sclerosis [56]. Moreover, the anti-inflammatory activity of *miR-*455 was also described by recent studies reporting an efficient therapeutic role of this miRNA in acute liver injury and cerebral ischemia/reperfusion injury [57, 58]. Another interesting result is *miR-*361 upregulation, which seems to be involved in the regulation of biological processes related to the immune response. In bovine, the level of *miR-*361 has been reported to be lower in the serum of grazing cows than in that of housed cattle [59]. This study may suggest a role for *miR-*361 in the regulation of metabolism in cows. It is well known that mastitis affects cow metabolism by altering lipolysis and the milk proteome [60, 61]. Therefore, the upregulation of *miR*-361 in our samples may depend on metabolic factors modified by mastitis. In addition, in humans, *miR-*361 is reported to be a promising biomarker for tuberculosis diagnosis and is most likely involved in the regulation of host‒pathogen interactions [62, 63]. The downregulation of *miR-*1301 was first reported in mastitis in this study. Luoreng and colleagues reported the upregulation of *miR-*1301 in the blood of dairy cows experimentally infected with a *Staphylococcus aureus* strain [64]. This controversial result may be partially explained by the different biological fluids analysed, the milk used in the present study and the blood used in the previous study. Furthermore, *S. aureus* was never identified in the milk samples of this study. It is well known that etiological agents can influence mastitis pathogenesis differently [26, 64], and differences in the milk miRNome between mastitis caused by either *Escherichia coli* or *S. aureus* have already been reported [65]. Therefore, the differential regulation of *miR-*1301 depending on the specific pathogen could not be excluded. Finally, *miR-*503 represents another important node of regulation according to the functional analysis. Most information regarding *miR-*503 is related to human diseases. This miRNA seems to be involved in the pathogenesis of diabetes and lipopolysaccharide injury [66, 67], but more intriguingly, its downregulation has been reported to be involved in the activation of NF-kB signalling and the PPARγ pathway [68, 69]. In veterinary medicine, the downregulation of *miR*-503 has also been reported in dog blood mononuclear cells infected with *Leishmania infantum*, suggesting a role in the modulation of the host‒response [70].

One innovative aspect of this study is the selection of dairy cows naturally affected by mastitis. Studies with an in-field scenario allow the evaluation of pathological conditions while taking into account the actual environmental variability. Moreover, some studies, even if conducted on naturally infected cows, considered a very low sample size [71, 72] in comparison with the sample size in this study (60 Holstein Friesian cows). Considering the method applied, another important consideration can be made. Small RNA-seq allows the investigation of the wide and complete panorama of miRNA expression and activity. On the other hand, studies applying qPCR or microarrays can focus only on selected and limited miRNAs and do not allow the identification of novel bovine miRNAs [49, 73, 74]. Finally, the other two studies, from Bagnicka et al. and Özdemir, represent the most comparable studies with the present study [75, 76]. Similar methods for small RNA-seq and a smaller (but still well representative) sample size have been applied. However, the main DE miRNAs reported are different from those identified in the present study. The main reason is likely related to the animal selection modality, which analysed only dairy cows positive for coagulase +/-*Staphylococci* or *M. bovis*. Instead, the present study focused on the selection of cows on SCC and not on bacteriological results. Furthermore, as already mentioned, the different biological matrices could influence the results. In fact, the origin of miRNAs can affect the miRNA profile. For example, whole milk has a different miRNA profile than skim milk, somatic cells, milk fat globules, or mammary gland biopsies [77–79].

In conclusion, the results obtained from this study could be a promising starting point for the investigation of miRNA and EV regulation in bovine subclinical mastitis. The functional analysis revealed a panel of four miRNAs that are promising putative biomarkers of subclinical mastitis: *miR*-455, *miR-*361, *miR-*1301 and *miR*-503. Notably, these results were validated via predictive analysis using human orthologues; therefore, their role may not be the same in bovines. To clarify the DE miRNA results, future validation via qPCR or ddPCR may be performed on other dairy cows. Furthermore, to define the role of DE miRNAs in the regulation of genes identified by functional analysis and therefore in inflammation, an in vitro luciferase reporter gene assay to validate miRNA target sites in bovines may also be performed. Nevertheless, the poor presence of mastidogen bacteria observed in the dataset may represent a limitation of this study, and an additional validation of the obtained results may involve the inclusion of gram-negative or *Mycoplasma* spp. mastitis forms. Further studies must be conducted to identify and validate miRNAs used for mastitis detection, especially subclinical forms. These methods can be considered integrative approaches among clinical evaluation, SCC, microbiology and miRNAs, which will always constitute the optimal strategy for early detection of bovine mastitis.

## Supplementary Information

**Additional file 1: PCA table for sequenced miRNAs.**

**Additional file 2: DE miRNA list**. In Additional file 1, the list of DE miRNAs with related FC and p value data is reported, with an SCC cut-off of 200,000 cells/mL used for differential analysis.

## Data Availability

The dataset(s) supporting the conclusions of this article is (are) available in the Genome Sequence Archive (GSA) repository, identified with the code *subCRA018425* at https://ngdc.cncb.ac.cn/gsub/submit/biosample/list.
