# Supplementary figures and images for: Extracellular vesicle miRNome during subclinical mastitis in dairy cows
Source: Vet Res. 2024 Sep 19;55:112. doi: 10.1186/s13567-024-01367-x (PMC11414160; doi:10.1186/s13567-024-01367-x)

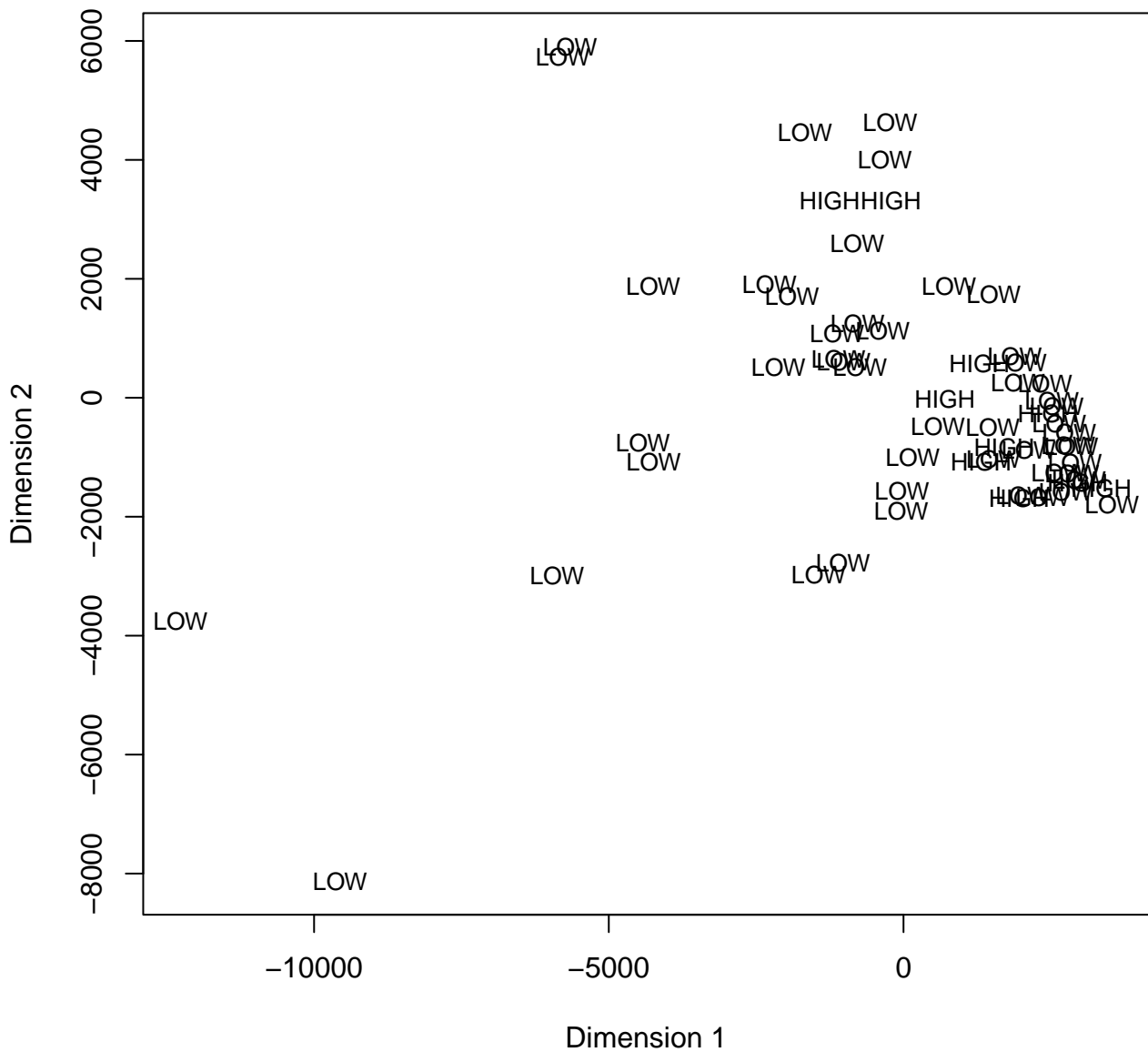

Supplement: Supplementary file 1 — Additional file 1: PCA table for sequenced miRNAs. [file 13567_2024_1367_MOESM1_ESM.pdf]
